# Characterisation and Sensitivity of a Canine Mast Cell Tumour Line to Oncolytic Viruses

**DOI:** 10.1111/vco.13024

**Published:** 2024-11-11

**Authors:** Yeganeh Mehrani, Julia E. Kakish, Christina Napoleoni, Jennifer Jane Thompson, Jason P. Knapp, Jessica A. Minott, Jacob G. E. Yates, Deirdre Stuart, Brenda L. Coomber, Robert A. Foster, Byram W. Bridle, Khalil Karimi

**Affiliations:** ^1^ Department of Pathobiology, Ontario Veterinary College University of Guelph Guelph Ontario Canada; ^2^ Companion Animal Tumour Bank, Ontario Veterinary College University of Guelph Guelph Ontario Canada; ^3^ Department of Biomedical Sciences Ontario Veterinary College, University of Guelph Guelph Ontario Canada

**Keywords:** cancer, canine mast cell tumours, oncolytic virotherapy, oncolytic viruses

## Abstract

Canine mast cell tumours (MCTs) are one of the most common skin cancers of dogs. Surgical removal is the primary treatment, but recurrence and metastasis can occur even with low‐grade tumours. As a result, new treatment strategies are being sought. We tested the potential of several oncolytic viruses (OVs) to infect and kill a cell line isolated from a canine MCT. Employing a resazurin‐based metabolic assay and flow cytometry technology, we used recombinant vesicular stomatitis virus (rVSV‐Δm51), avian orthoavulavirus‐1 (AOaV‐1), and Orf viruses in our assessment. Our study aimed to evaluate the potential of oncolytic virotherapy in treating canine cancers. We found that MCT‐1 cells showed different sensitivities to the OVs, with rVSV‐Δm51 showing the most promising results in vitro. These findings suggest that further investigation into using OVs for treating canine MCTs is needed, although clinical efficacy is yet to be determined.

## 1 Introduction

Cancers are a common cause of nontraumatic death of dogs [1], and mast cell tumours (MCTs) are particularly prevalent [2]. In dogs, mast cell tumours can occur at any age, with the diagnosis occurring on average around 8 years [3]. The standard of care for treatment of tumours includes surgery, radiation therapy, and chemotherapy [4]. The treatment of MCTs in dogs and cats depends on tumour location, grade, and stage. Surgical excision is the primary approach when the tumour can be entirely removed. If surgical margins are inadequate or the tumour shows aggressive features according to the 2‐tier histologic grading system proposed by Kiupel et al., further intervention such as additional surgery, radiotherapy, or chemotherapy may be necessary [5]. If local control cannot be achieved or if there is metastasis, chemotherapy is the recommended supportive care [3, 6].

Several new therapeutic techniques in human oncology have been created and are being tested on pets. One new technique is oncolytic virotherapy [7]. Oncolytic viruses (OVs) represent a novel type of cancer treatment that uses multiple mechanisms to fight the disease. Over the last two decades, researchers have made substantial progress in understanding the mechanisms OVs employ against cancer cells [8, 9, 10]. These viruses work by infecting malignant cells and inducing lysis to promote infection of neighbouring tumour cells [11]. A hallmark of OVs is their specificity, allowing them to productively infect and kill cancerous cells with minimal impact on healthy cells [12]. This selectivity may be a naturally favourable characteristic of the virus used or due to genetic modifications incorporated into the virus [11]. An ideal virus must demonstrate potent oncolytic properties and target signalling pathways with unique expression profiles in certain cancers [13]. When OVs directly infect and destroy tumour cells [11], the antigens linked with the tumour cells are released. This provides the danger signals and target antigens needed to trigger a tumour‐specific immune response. Indeed, oncolytic virotherapy can be considered a therapeutic cancer vaccination method because it induces specific anti‐cancer immune responses. OVs' ability to preferentially infect, replicate in and kill tumour cells while leaving most healthy cells intact makes them an alternative and promising therapeutic approach to treating canine cancers. A published study showed that canine MCTs have variable sensitivities to the oncolytic effects of Sendai virus [14].

Recombinant vesicular stomatitis virus (rVSV‐Δm51) has been proven to be effective in a broad array of murine models of cancers [15, 16]. Evidence indicates that Orf virus, a species within the *Parapoxvirus* genus, is a promising candidate for treating cancers due to its unique immuno‐stimulatory properties and low pathogenicity outside its host species, sheep. Moreover, it causes anti‐cancer effects in various syngeneic mouse cancer models, with the mechanism of action primarily due to the potent activation of cytotoxic and cytokine‐secreting NK cells [17]. Avian orthoavulavirus‐1 (AOaV‐1) expressing immunoregulatory genes have been shown to kill cancer cells of various origins [18].

## 2 Materials and Methods

### 2.1 Viruses

This study utilised four OVs: vesicular stomatitis virus (VSV), ORFV, and two versions of AOaV‐1: the lentogenic and mesogenic strains. Dr. Andrew Mercer, University of Otago, kindly provided OrfV‐NZ2 (ORFV). OrfV was propagated and titrated by the TCID_50_ assay on sheep skin fibroblast cells (SSF cells) as described previously [19]. We made two versions of our proprietary AOaV‐1: lentogenic (l‐AOaV‐1) and mesogenic (m‐AOaV‐1). These designations are based on pathogenicity in poultry, where l‐AOaV‐1 and m‐AOaV‐1 are of low and high pathogenicity, respectively. In Canada, both are considered biocontainment Level 2 viruses. In other jurisdictions, such as the United States of America, m‐AOaV‐1 is considered a biosafety Level‐3 pathogen. We wanted to compare the two to see if l‐AOaV‐1 would perform as well as the m‐AOaV‐1, which would have advantages for cross‐border translation of the findings of this study. Two of our proprietary strains of m‐AOaV‐1 and l‐AOaV‐1 were utilised. Both are fully synthetic viruses that encode an enhanced green fluorescent protein transgene (AOaV‐1‐GFP) between the phosphoprotein (P) and matrix protein (M) genes, and both contain an L289A mutation in the fusion protein. The recombinant VSV carried a transgene encoding GFP. Dr. Brian Lichty kindly provided a highly attenuated version with a deletion at position 51 of the matrix protein (McMaster University, Hamilton, Ontario, Canada).

We sought to examine the susceptibility of the canine MCT cell line to the oncolytic effects of rVSV‐Δm51, AOaV‐1 and ORFV. First, we isolated, cultured and characterised an MCT cell line that was derived from a primary MCT. The purpose was to identify ideal OVs to carry forward into a clinical trial with dogs bearing MCTs.

### 2.2 Generation of a Neoplastic Canine Mast Cell Line (MCT‐1) Using Fibroblast Co‐Culture

Neoplastic mast cells were isolated from surgical biopsies [20, 21]. Surgical biopsies were obtained from a 7‐year‐old male castrated Sharpei dog diagnosed with a Grade III and high grade spontaneous MCT with metastasis to the mandibular lymph node. The small animal surgical oncology service at the Animal Cancer Centre, Ontario Veterinary College, University of Guelph provided these biopsies. The cell line was derived from a sample collected from a surgical specimen as part of the ICCI canine cancer tumour bank. Client consent was obtained for tumour banking. The approval of the client consent form for our tumour bank was vetted by the University of Guelph's legal office and ultimately signed off by the VP of Research. Cells were initially maintained in co‐culture with fibroblasts using suspension plates and modifications of published techniques [21, 22]. Roswell Park Memorial Institute (RPMI) medium (RPMI‐1640; Sigma‐Aldrich, Oakville, ON, Canada) was supplemented with 10% fetal bovine calf serum (FBS) (Avantor, USA), 1 mmol/L sodium pyruvate (Gibco, New York, USA), 100 U/mL penicillin (HyClone, South Logan, UT), 100 μg/mL streptomycin (HyClone, South Logan, UT), 50 μg/mL gentamycin (Gibco, New York, USA), 50 μg/mL amphotericin B (Invitrogen, Burlington, ON, Canada) and 10 ng/mL of recombinant canine stem cell factor (rcSCF; R&D Systems, Minnesota, United States). Every 3–5 days, the media and rcSCF were replenished with gentle pipetting to avoid removing adherent mast cells while allowing debris, erythrocytes and other leukocytes to be removed. Mast cells were initially suspended or adhered to fibroblasts. After a few weeks, a cell line designated ‘MCT‐1’ began to proliferate without rcSCF or fibroblasts. Wright–Giemsa‐stained cytospins were used to determine purity, and trypan blue dye exclusion was used to assess viability.

### 2.3 Maintenance Culture of MCT‐1 Cells

MCT‐1 cells were maintained in vitro in Dulbecco's modified Eagle medium (HyClone; South Logan, UT). All media were supplemented with 20% heat‐inactivated FBS (Avantor, USA) and 2 mL of Gentamicin (Gibco, New York, USA) per 50 mL total media volume. The cells were cultured in a humidified incubator at 5% CO_2_ and 37.0°C.

### 2.4 Assessment of Expression of P‐KIT and KIT by Immunofluorescent Microscopy

Cells were serum‐starved for 48 h prior to fixation with 4% paraformaldehyde. MCT‐1 cells were cultured on sterile coverslips. Following permeabilisation and blocking as above, slides were incubated with an Alexa Fluor 488‐conjugated mouse monoclonal antibody against KIT (Cell Signalling Technology; 1:50) overnight at 4°C. Slides were rinsed and incubated with rabbit anti‐p‐KIT (Cell Signaling Technology; 1:100) for 1 h at room temperature prior to incubation with a Cy3‐tagged rabbit‐specific secondary antibody (1:100; Jackson ImmunoResearch Laboratories, West Grove, PA, USA). Nuclei were stained using 4 ng/mL of 4′,6′‐diamidino‐2‐phenylindone (DAPI; Dako, Carpinteria, CA, USA), and slides were mounted using fluorescence mounting media (Dako, Carpinteria, CA, USA). Images were captured with a 40× objective using QCapture software calibrated to a Leica DMLB microscope fitted with a Q imaging QICAM fast 1394 digital camera. Images were merged using Adobe Photoshop 7 (Adobe).

### 2.5 Immunocytochemical Expression and Activity of KIT and Vascular Endothelial Growth Factor Receptor‐2 (VEGFR2)

Cells were serum‐starved for 48 h. To detect phospho‐VEGFR2 (p‐VEGFR2), cells were incubated with 0.5 mM sodium pervanadate for 8 min at 37°C prior to experiments. Air‐dried cytospins were prepared using approximately 1 × 10^6^ cells/mL resuspended in phosphate‐buffered saline (PBS; Hyclone, South Logan, UT) prior to fixation with 4% paraformaldehyde. Cells were permeabilised using 0.1% Triton X‐100 (Sigma‐Aldrich, Oakville, ON, Canada) for 5 min, rinsed with wash buffer (Dako, Carpinteria, CA, USA), blocked with protein‐free blocking solution for 10 min (Dako), followed by incubation with primary rabbit antibodies specific for KIT (Dako; 1:100; phospho‐KIT [p‐KIT] and VEGFR2 [all from Cell Signalling Technology; used at 1:100]) for 1 h at room temperature. A LSAB Kit (Streptavidin‐biotin‐HRP Labelling Kit for Immunohistochemistry) (Dako, Carpinteria, CA, USA) was used as per the manufacturer's protocol using 30‐min incubation times for anti‐rabbit biotinylated antibodies and peroxidase‐labelled streptavidin. Reactions were visualised with 3′,3′‐diaminobenzidine (DAB; Dako, Carpinteria, CA, USA). Following haematoxylin counterstaining, slides were mounted (Cytoseal; Thermo‐Scientific; Nepean; ON, Canada) and coverslipped.

### 2.6 Cell Viability Assays

Resazurin assays were conducted to quantify the metabolic activity of MCTs, which correlates with cell viability. This was done to evaluate the oncolytic potential of viruses in this cell line. There were control wells containing only media and other control wells with only cells without virus treatment. MCT‐1 cells were seeded at 8458 cells/well, which was deemed optimal after conducting a pilot cell seeding assay to ensure that cells would not overgrow. The MCT‐1 cells were incubated for 24 h before treatment with viruses to allow the cells to adhere. The MCT‐1 line was treated with nine different multiplicities of infection (MOI) of rVSV‐Δm51, ORFV and two strains of AOAV‐1 (m‐AOAV‐1 and l‐AOAV‐1). The cells were treated with a virus at a range of MOI from 10 to 0.0065 (2.5‐fold serial dilution) and incubated for 48 h (It is important to note that an MOI of 10 is very high and typically unachievable in a clinical setting). After 48 h, resazurin sodium salt (Sigma‐Aldrich, Oakville, ON, Canada) was added at a final concentration of 0.25 mg/mL, and fluorescence was quantified with a plate reader (excitation wavelength: 535/25 nm, emission wavelength: 590/35 nm) 4 h later. Cells still metabolically active were pink in colour, while dying cells were blue. In living cells, the blue resazurin dye was converted to pink resorufin. The level of fluorescence dropped as cancer cells were killed; the amount of fluorescence was quantified with the plate reader. Cell viability was calculated as a percent change relative to untreated control cells after subtracting the background fluorescence of the wells containing media only.

### 2.7 Expression of GFP in MCT Cells Treated With OVs

Flow cytometry was utilised to evaluate the expression of GFP in MCT cells infected with OVs. On Day 1, MCT‐1 cells were seeded in 96‐well plates at 8300 cells/well and incubated for 24 h to allow the cells to adhere. On Day 2, the MCT‐1 cells were treated with the OVs at an MOI of 10. The plates were incubated for 24 and 48 h, after which the wells were prepared to run on a flow cytometer. First, the MCT‐1 cells were centrifuged at 1000× *g* for 5 min to avoid losing too many dead cells. The media were decanted, and the cells were resuspended with PBS and centrifuged at 500× *g* for 5 min. The MCT‐1 cells were then resuspended using 10 mM ethylenediamine tetraacetic acid (EDTA) and incubated for 10–15 min to detach the remaining adhered cells. EDTA was used to remove adhesion molecules without destroying the surface molecules of the cells. The cells were then centrifuged at 500× *g*, the media were decanted, and cells were resuspended in PBS. This wash step was repeated twice. After the second wash step, the cells were resuspended by tapping the plate, and a 7‐aminoactinomycin D (7AAD) viability marker (Bio Legend; San Diego, CA, USA) was added to the wells (5 μL of 7AAD and 195 μL of fluorescence‐activated cell scanning [FACS] buffer [PBS + 0.5% bovine serum albumin; Hyclone, South Logan, UT]) per well, and incubated at 4.0°C for 20 min. Two more wash steps followed this, and then the samples were resuspended in FACS buffer before running on a FACS Canto II flow cytometer (BD; San Jose, CA).

### 2.8 Plaque Assay to Quantify Infectious Virions

MCT‐1 cells (750 000 cells per well) were treated with rVSV‐Δm51 at MOIs of 0.1 and 10. Virus‐containing supernatants were collected at 0, 8, 16, 24, 32, 40, and 48 h post‐infection, and the quantity of replication‐competent virions in the supernatant was evaluated using a standard plaque assay. One day prior to infection, 350 000 Vero cells (American Type Culture Collection, USA) were seeded into each well of a six‐well tissue culture plate using DMEM (Cytiva, USA) containing 10% FBS. On the day of infection, virus‐containing supernatants were thawed at room temperature, and serial 10‐fold dilutions were prepared in DMEM containing 10% FBS. The media in the wells were discarded, and the cells were washed with 1× PBS and then infected with 500 μL of the diluted supernatants. Monolayers were infected for 1 h at 37°C with gentle rocking every 5 min to allow for efficient spreading and adsorption of virions. Following the infection, the inoculum was removed, and monolayers were washed with 1 mL of 1× PBS. The cells were then overlaid with 3 mL of a plaquing mixture, which contained DMEM‐10% FBS, 2× minimum essential medium (MEM; Corning, New York) and 2% agarose (Invitrogen, USA) at a ratio of 2:1:1. Once the overlay solidified, plates were incubated at 37°C and 5% CO_2_, and then the number of virus‐induced plaques was counted 24 h later. Viral titres, as a measure of plaque‐forming units per millilitre, were calculated using the following formula: Viral titre = (Average number of plaques)/(Dilution × Volume of diluted sample added to plate in μL).

### 2.9 Statistical Analyses

GraphPad Prism 9 (GraphPad Software, San Diego, California, USA) was used for all graphing and statistical analysis. Metabolic resazurin data, which involved two variables, were assessed by two‐way analysis of variance with Dunnett's multiple comparisons test. All reported *p* values were two‐sided and were considered significant at *p* ≤ 0.05.

## 3 Results

### 3.1 Identification and Establishment of an Adherent Neoplastic Canine MCT‐1 Line

**FIGURE 1:**
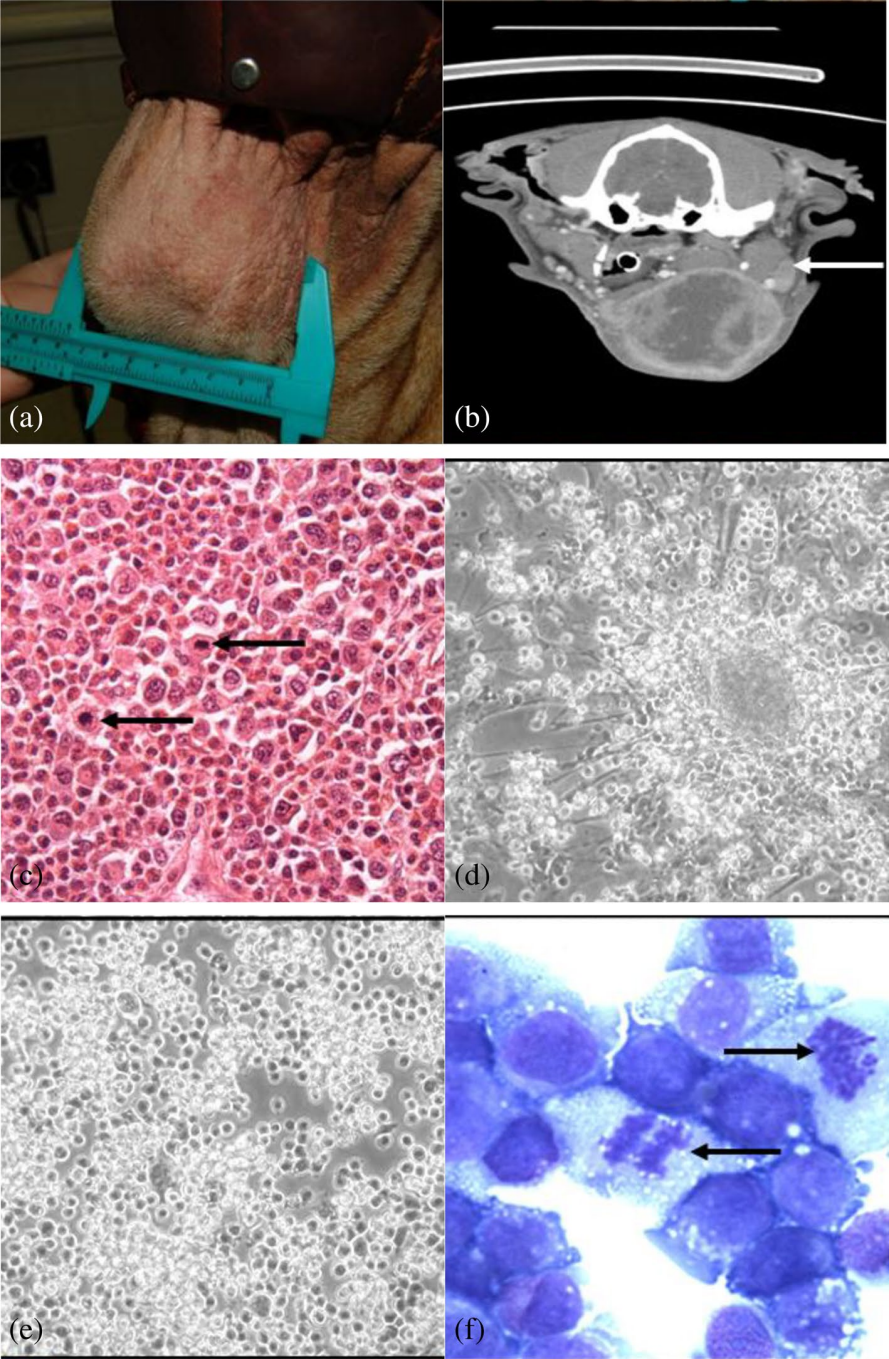
Isolation of a pure population of mast cells with cytoplasmic granules and numerous mitotic features. (a) A cell line called ‘MCT‐1’ was isolated from a metastatic cutaneous mast cell tumour (MCT) on the neck of a 7‐year‐old male castrated Sharpei dog. (b) Computed tomography image of the tumour showing metastasis to the local submandibular lymph node. (c) Histology of the original tumour (Grade III) is shown. The tumour was composed of a densely cellular pleomorphic population of agranular mast cells. Cells had distinct borders, central round, oval, convoluted and frequently multiple nuclei, indistinct nucleoli, 3‐fold anisocytosis and anisokaryosis and a mitotic index of 55 per 10 high power fields (arrows) (400X, HE). (d) MCT‐1 grew as adherent spheroid colonies, initially attached to fibroblasts (200×) and (e) proliferated without fibroblast attachment after 3 months in culture (200×). (f) Cytospin shows a pure population of MCs with cytoplasmic granules and numerous mitotic features (arrows) (Wright–Geimsa; 1000×).

A high grade and Grade III MCT were obtained from the neck of a 7‐year‐old male castrated Sharpei dog with metastases to the mandibular lymph node (Figure 1a,b). The tumour was a non‐encapsulated, poorly demarcated expansile and infiltrative mass extending from the dermis to the subcutaneous tissue, composed of a dense cellular, pleomorphic population of agranular neoplastic mast cells (Figure 1c). The cells had distinct borders, central round, oval, convoluted, and frequently multiple nuclei, indistinct nucleoli, three‐fold anisocytosis, and anisokaryosis, and a mitotic index of 55 per 10 high power fields (Figure 1c, arrows). Culturing with fibroblasts, the MCT‐1 cell line grew as adherent spheroid colonies, initially attached to fibroblasts (Figure 1d) that proliferated without fibroblast attachment after 3 months in culture (Figure 1e). Cytology of a cytospin showed a pure population of MCT‐1 cells with cytoplasmic granules and numerous mitotic features (Figure 1f). The pure mast cell population was stained with Wright–Giemsa cytospin (Figure 1f).

### 3.2 The MCT‐1 Cell Line Expressed Activated KIT and VEGFR2

Specific features were assessed to characterise the MCT‐1 cell line, including auto‐phosphorylated KIT and VEGFR2, which are prognostic factors whose expression can increase without exogenous ligation.

The antibodies used in our study were validated for specificity and cross‐reactivity prior to experimentation. Immunoprecipitation confirmed specificity, ensuring accurate detection of KIT and VEGFR2 without cross‐reactivity. Each experiment included appropriate positive and negative controls. The canine cerebellum was used as a positive control for KIT and phosphorylated KIT, while the canine aorta served as the positive control for total and phosphorylated VEGFR2. Canine soft tissue sarcoma, which does not express these proteins, was used as a negative control to confirm the specificity of antibody staining.

**FIGURE 2:**
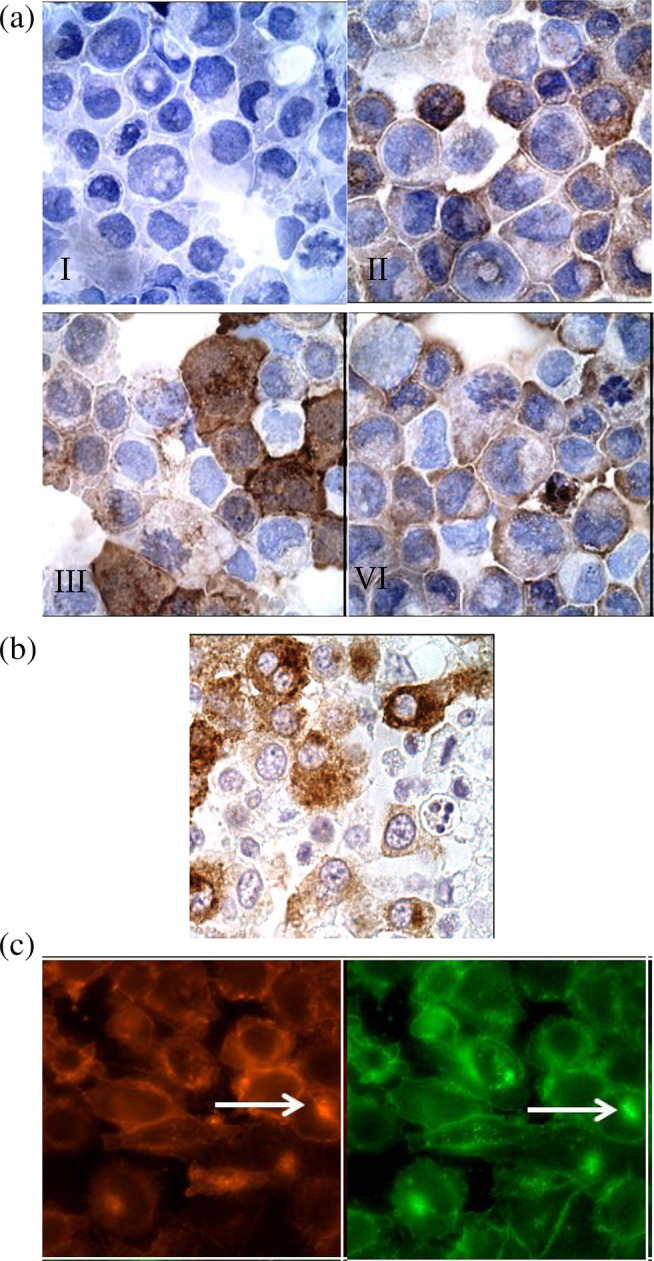
Immunocytochemical and immunofluorescent localization of phosphorylated and total KIT and VEGFR2 in MCT‐1 cells. (a) MCT‐1 cells displayed heterogeneous phosphorylation of KIT and expression of VEGFR2 detected by immunocytochemical analysis. A coarse dark brown cytoplasmic stain was observed in scattered cells, varying in intensity from stippled to diffusely cytoplasmic. The panels are shown as I. Negative control (no primary antibody), II. Total KIT expression (using anti‐KIT antibody), III. Phospho‐KIT (p‐KIT) expression (using anti‐p‐KIT antibody) and IV. VEGFR2 expression (using an anti‐VEGFR2 antibody). (b) Immunohistochemical assessment was performed to evaluate KIT localization in MCT‐1 cells, revealing diffuse coarse brown cytoplasmic staining for KIT in approximately one‐third of the cells using an anti‐KIT antibody. (c) The cellular localization of phosphorylated and total KIT in MCT‐1 cells was examined using immunofluorescent microscopy. Serum‐starved cells were stained with an anti‐KIT antibody (green) and an anti‐p‐KIT antibody (Tyr 719; red). The results showed predominantly stippled and focal (‘Golgi‐like’) cytoplasmic expression and phosphorylation of KIT, indicated by an arrow. Additionally, weak membranous expression of total KIT was observed. The magnification used for the examination was 1000×.

The expression of KIT and VEGFR2 was observed in most MCT‐1 cells, detected by diffuse stippled cytoplasmic staining (Figure 2a). Cell‐to‐cell heterogeneity was also apparent in the phosphorylation of KIT and expression of VEGFR2 as assessed by immunocytochemistry. Immunohistochemistry was utilised to assess the localisation of KIT in MCTs, which were identified by a characteristic coarse brown staining pattern (Figure 2b). Additionally, immunofluorescence microscopy showed diffuse and focal perinuclear (‘Golgi‐like’) co‐localisation of KIT and p‐KIT (Figure 2c).

In MCTs, *c‐Kit* is a known requirement for tumour cell survival [23, 24]. We cultured and phenotypically characterised cancerous cells isolated from a 7‐year‐old male castrated Sharpei dog. This cell line was used to measure the expression of the key prognostic markers including tyrosine‐protein kinase Kit (CD117) [23], VEGFR2, confirming their origin from a mast cell tumour and suitability for studying as targets for OVs.

### 3.3 Evaluating the Potential to Treat MCT‐1 Cells With OVs

**FIGURE 3:**
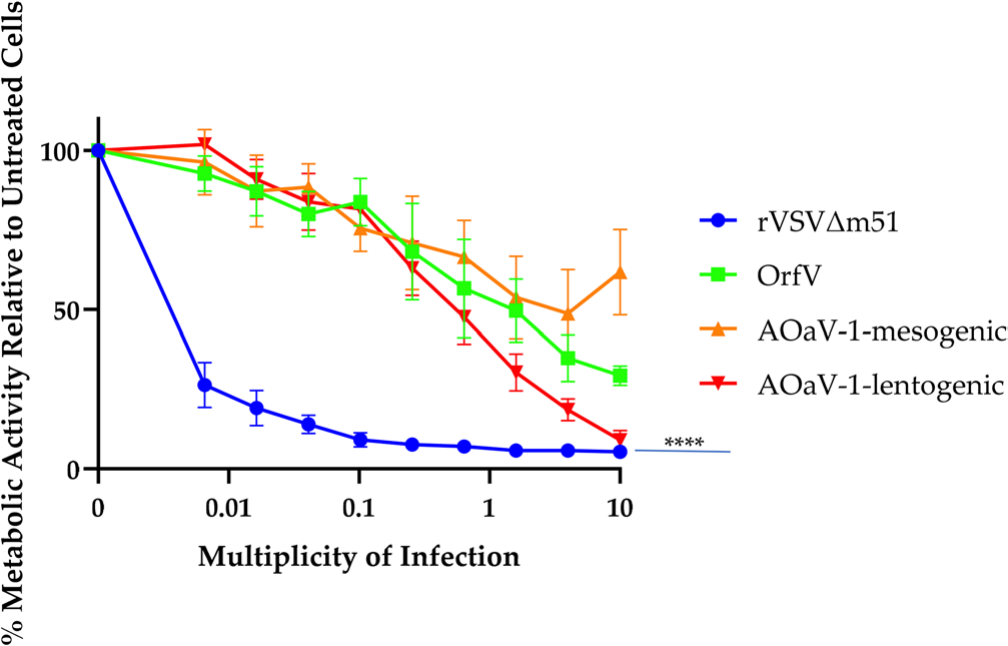
Differential viral oncolytic efficacy in a cell line derived from a MCT‐1. (a) A dose–response graph showing the cytotoxicity of rVSVΔm51, ORFV, mesogenic AOaV‐1, and lentogenic AOAV‐1 in MCT‐1 cells. Cytotoxicity was assessed using a metabolic resazurin dye assay. The error bars represent the standard error of the mean (SEM). Statistical analysis was performed using a two‐way analysis of variance with Tukey's multiple comparison test to compare the overall mean column effect among the treatment groups. *p* values for the comparisons between oncolytic viruses are shown (*n* = 3/treatment group).

To determine the susceptibility of MCT‐1 cells to OVs, a resazurin dye‐based assay was used to examine cell metabolic activity as a proxy for viability following treatment with the viruses rVSVΔm51, ORFV, and m‐AOaV‐1 and l‐AOaV‐1. All viruses had an oncolytic effect that correlated with dose, with rVSVΔm51 being, by far, the most potent OV (*p* < 0.0001) (Figure 3, Table S1). Notably, the l‐AOaV‐1 was significantly more oncolytic than the m‐AOaV‐1 (*p* = 0.0044).

### 3.4 Vulnerability of MCT‐1 Cells to Infection After Treatment With OVs

**FIGURE 4:**
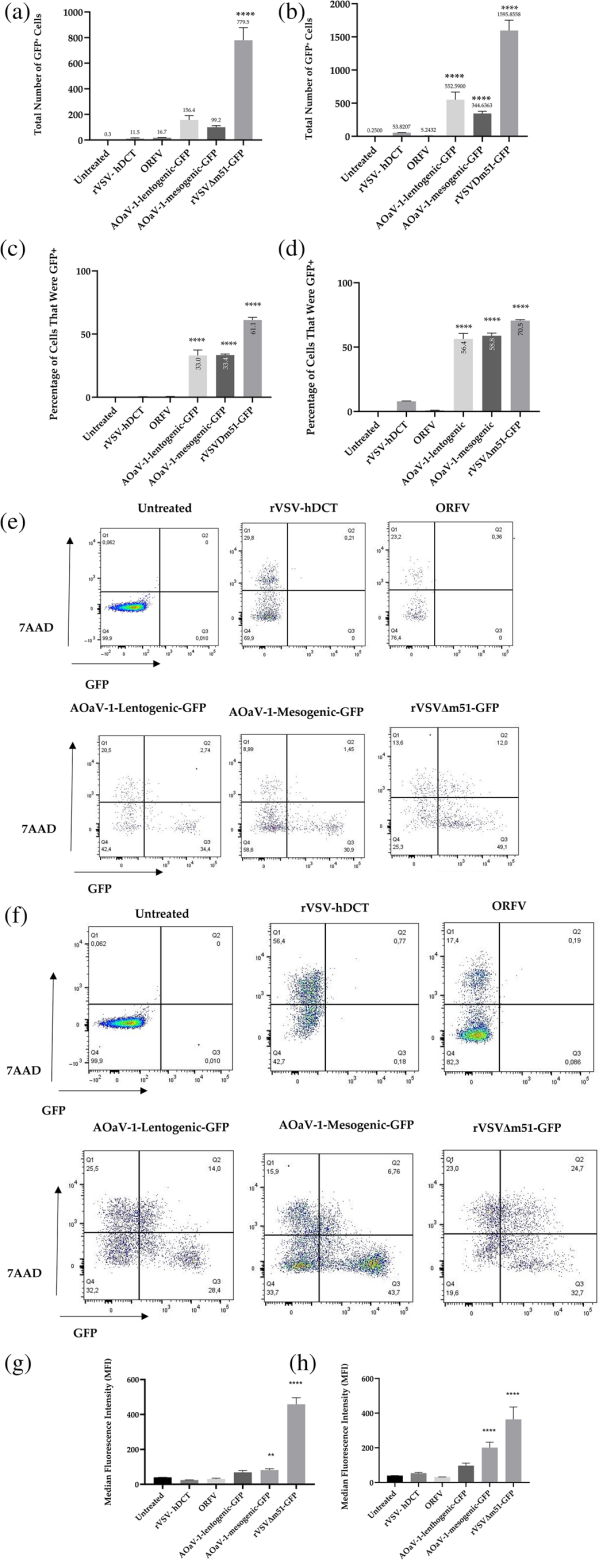
Transgene expression and cytotoxicity mediated by oncolytic viruses in a cell line derived from a canine mast cell tumour (MCT‐1). Flow cytometry was used to detect GFP following challenge of the MCT‐1 cells with a panel of oncolytic viruses (rVSV‐hDCT, ORFV, AOaV‐1‐mesogenic‐GFP, AOaV‐1‐lentogentic‐GFP or rVSVΔm51‐GFP). Total number of GFP‐positive cells at (a) 24 and (b) 48 h post‐infection. The error bars depict the mean and standard deviation (SD) (****p < 0.0001). Percentages of total cells that were GFP‐positive at (c) 24 and (d) 48 h post‐infection (****p < 0.0001 compared with untreated cells.). Representative dot plots showing expression of GFP versus staining with 7AAD from two replicate experiments at (e) 24 and (f) 48 h after treatment. Median fluorescence intensity of GFP expression was determined by flow cytometry at (g) 24 and (h) 48 h after treatment (****p < 0.0001, **p = 0.0076). Statistical analyses were done by one‐way analysis of variance and Tukey's multiple comparison tests were used to define statistical significance as *p < 0.05.

OV‐mediated transgene expression in MCT‐1 cells was assessed by flow cytometry. Specifically, MCT‐1 cells were treated at a MOI of 10 with a rVSV expressing a non‐fluorescent protein (human dopachrome tautomerase) (rVSV‐hDCT), ORFV, m‐AOaV‐1, l‐AOaV‐1 or rVSVΔm51, the last three encoding the full‐length transgene for enhanced green fluorescent protein. Controls included uninfected cells and cells treated with a rVSV‐hDCT and ORFV. As shown in Figure 4a–d, both AOaV‐1 viruses and rVSVΔm51 could mediate significant transgene expression. A cell viability dye was applied to the cells prior to flow cytometry analysis to differentiate live and dead MCT‐1 cells. As shown in Figure 4e–h m‐AOaV‐1, l‐AOaV‐1 and rVSVΔm51 produced significant amounts of GFP in cells.

### 3.5 rVSVΔm51 Infections in MCT‐1 Cells Were Productive

**FIGURE 5:**
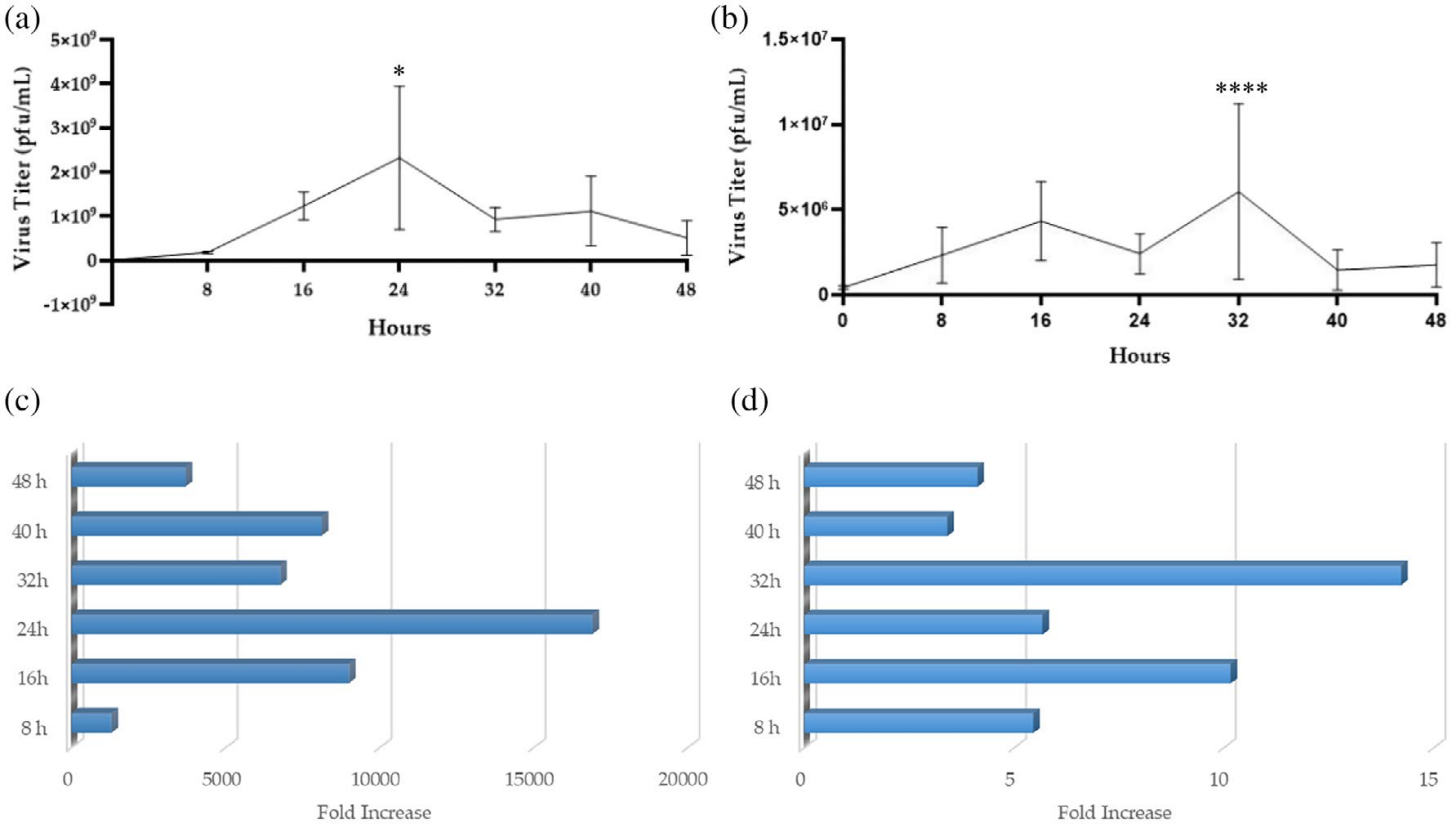
rVSVΔm51 mediated a productive infection in a cell line derived from a MCT‐1. MCT‐1 cells were treated with rVSVΔm51 for 0, 8, 16, 24, 32, 40 and 48 h. The supernatants from cells treated at a multiplicity of infection (MOI) of (a) 0.1 or (b) 10 were used to quantify infectious virions using plaque assays. Statistical analysis was performed using a one‐way analysis of variance and Tukey's multiple comparisons test. Means ± standard errors for rVSVΔm51 titres are depicted (*n* = 3; (a) *p < 0.03, (b) ****p < 0.0001). The fold‐increases in viral titres compared with the input dose are shown for MCT‐1 cells treated at a MOI of (c) 0.1 and (d) 10.

We demonstrated that rVSVΔm51 could infect, mediate transgene expression and kill MCT‐1 cells. However, we wanted to know if this infection resulted in the amplification of the virus, which is an important clinical feature to facilitate the spreading of the virus through a tumour. We concentrated on rVSVΔm51, since it was the most effective of the OVs throughout all assays. A standard plaque assay was used to quantify the number of replication‐competent virions. The assay counts the number of plaque‐forming units (pfu) formed in a monolayer of virus‐infected cells and assesses the ability of the virus to productively infect cancer cells. MCT‐1 cells were infected with rVSVΔm51 at MOIs of 0.1 or 10. As shown in Figure 5, both doses resulted in productive infections, with > 15 000‐fold and > 14‐fold increases in titres at MOIs of 0.1 and 10, respectively. However, a far higher peak titre was achieved with a starting MOI of 0.1 (~1 × 10^9^ pfu/mL) as compared with an MOI of 10 (only ~5 × 10^6^ pfu/mL).

## 4 Discussion

MCTs can exist as an isolated mass, invade local tissues or metastasize to lymph nodes and other organs such as the spleen, liver, gut and bone marrow [25]. Since current standard of care treatments for MCTs, such as surgery, may result in insufficient clearance of cancer cells, other therapies may be more effective. Oncolytic virotherapy is one of these methods that may target and kill MCT‐derived cells anywhere in the body. The current study evaluated the histological features of a canine MCT‐derived cell line, which confirmed that it originated from mast cells. This cell line was then used to evaluate the potential of different OVs to be used as therapeutic alternatives for these kinds of cancers.

The first objective of this study was to investigate the potential of the OVs ORFV, AOaV‐1 and rVSVΔm51 to kill MCT‐1 cells. The rVSVΔm51s had the most significant oncolytic potential among the OVs that were examined. This is likely due to its rapid replication cycle relative to the other viruses [26]. Interestingly, the l‐AOaV‐1 was at least as effective as the m‐AOaV‐1.

The initial assay used to assess the oncolytic activity of the viruses was based on quantifying cellular metabolism as a proxy for cell viability. Therefore, the second objective was to assess whether the oncolytic activity of the viruses was due to infection of MCT‐1 cells and to confirm that this resulted in cell death. Flow cytometric assessments of virus‐mediated transgene expression confirmed that the MCT‐1 cells were getting infected and that a representative gene from the OVs AOaV‐1 and rVSVΔm51 was significantly expressed. The rVSVΔm51 mediated the greatest transgene expression. Again, there was an interesting finding with the AOaV‐1, whereby both the l‐AOaV‐1 could mediate transgene expression that was at least comparable with the m‐AOaV‐1.

The final objective of this study was to quantify replication‐competent virions in infected MCT‐1 cells, focusing on rVSVΔm51 as the most oncolytic of the tested viruses. A plaque assay showed that the rVSVΔm51 could proliferate up to > 15 000‐fold in MCT‐1 cells. Our findings suggested that treating MCT‐1 cells with the lower MOI of 0.1 resulted in an almost two‐log higher peak titre compared with an MOI of 10. This is ideal for the in vivo application of this virus as an oncolytic agent because it is almost impossible to achieve high MOIs in a clinical setting unless directly injecting a virus into a tumour, and even then, only a minimal number of cells will be exposed. The potential to achieve a high titre despite only a few cells initially getting infected would be expected to translate into the efficient spreading of a virus throughout a susceptible tumour.

Our study focused on examining the effects of OVs in a laboratory setting. It is important to consider our findings in the broader context of OV research, specifically its potential in treating canine tumours. These insights go beyond the limitations of our experimental setup. The direct application of the oncolytic adenovirus ICOCAV15 within epithelial cell tumours in dogs demonstrated the safety and effectiveness of this method, revealing a notable immunological reaction in the treated dogs. These findings highlight the potential of OVs to treat tumours in a clinical setting [27].

Furthermore, the therapeutic potential of OVs is further improved by research that examines the systemic delivery of the oncolytic vaccinia virus through canine adipose‐derived mesenchymal stem cells (cAdMSCs) in a xenograft mouse model. This study showed a significant reduction in tumour growth, which is a convincing illustration of how in vivo models might confirm the potential therapeutic effects reported in vitro [28].

Our research provides a fundamental understanding of the cellular impacts of OVs and demonstrates their potential for therapeutic use in clinical settings. Integrating laboratory‐based research and research conducted in vivo is crucial for advancing OV therapy in veterinary medicine.

There is a limited amount of literature discussing the potential of oncolytic virotherapy for treating canine mast cell tumours [29, 30]. Ilyinskaya et al. conducted a pilot study to determine whether OVs were safe and effective in treating MCTs in dogs. Their findings suggested that using the Sendai virus to treat MCTs in dogs could be effective. They also recommended more research to determine the best methods and regimes for this type of therapy [14]. Our study only compared four OVs. Future research could compare other previously characterised viruses such as measles, Semliki Forest, canine parvovirus serotype‐2 and myxoma [29, 31, 32, 33]. We envision a clinical future where oncology services could have access to multiple OVs to tailor treatments based on differential sensitivities of cancers and to facilitate heterologous multi‐dosing treatment regimens.

Furthermore, the success of oncolytic virotherapy for MCT treatment may depend on various factors, including the host's immune response, the location and stage of the tumour and the viral dose and route of administration. As a result, translational studies are needed to optimise the use of OVs for MCT treatment. Furthermore, combining oncolytic virotherapy with other therapies, such as chemotherapy and immunotherapy, may improve treatment efficacy and reduce the likelihood of cancers becoming resistant.

One limitation of the current study is the lack of non‐OV control, which would have been more appropriate to distinguish between the effects of viral infection and oncolysis. Including a non‐OV would have made it easier to determine whether the observed effects were caused by the viral infection or the virus's special oncolytic features. This limitation should be considered when interpreting the study's results, and future research should include non‐OV controls to enhance the understanding of OV specificity and therapeutic potential.

Concerning the panel of OVs evaluated in this study, the in vitro findings suggested that rVSVΔm51 was the best. However, rapid oncolysis due to a short replication cycle does not always translate into a virus being the most effective in vivo. In fact, there is some speculation that slower replicating viruses may hold some advantages in terms of the induction of tumour‐associated immune responses [34]. As such, the potential of ORFV and AOaV‐1 for clinical applications in the context of MCTs cannot be overlooked.

## 5 Conclusions

OVs may be a therapeutic option for MCTs. Early studies, albeit few in number, show encouraging results. Here, we have provided evidence that rVSVΔm51 could rapidly mediate a potent oncolytic effect, along with the promotion of transgene expression in an MCT cell line. Furthermore, ORFV and AOaV‐1 could also mediate oncolytic effects, but only the latter was particularly good at expressing a transgene. It is of particular interest that the much safer l‐AOaV‐1 was at least as efficacious as the m‐AOaV‐1 both in terms of oncolysis and transgene expression. This makes the l‐AOaV‐1 particularly attractive as a potential treatment for MCTs in jurisdictions that categorise m‐AOaV‐1 as a biological safety Level‐3 pathogen. The lentogenic version also abrogates the risk that dogs treated with this virus, especially those living on farms, could spread a pathogen to poultry. The rVSVΔm51, l‐AOaV‐1, and possibly ORFV could represent promising OVs for treating MCTs in dogs.

## Author Contributions

Conceptualization: K.K. and B.W.B. Methodology: Y.M., K.K., J.T., C.N, J.M., J.Y., D.S. Formal analysis: Y.M., J.K., and K.K. Investigation: Y.M. J.E.K., J.T., and K.K. Resources: K.K., B.L.C., and B.W.B. Writing – original draft preparation: Y.M. J.T., and J.E.K. Writing – review and editing: K.K., B.W.B., B.L.C., R.A.F., and Y.M. Supervision: K.K., B.W.B., and B.L.C. Project administration: K.K., B.W.B., and B.L.C. Funding acquisition: K.K. All authors have read and agreed to the published version of the manuscript.

## Ethics Statement

This research was approved by the University of Guelph's Animal Care Committee via Animal Utilisation Protocol # 4409. The approval of the client consent form for our tumour bank was vetted by the University of Guelph's legal office and ultimately signed off by the VP of Research.

## Consent

The authors have nothing to report.

## Conflicts of Interest

B.W.B. is the Chief Executive Officer of ImmunoCeutica Inc. (ICI), which is dedicated to the research and development of immunoceuticals. B.W.B. serves as a scientific advisor for the Canadian COVID Care Alliance (CCCA) Taking Back Our Freedoms (TBoF). Neither ICI, CCCA, nor TBoF were involved in any way with this manuscript and the research it describes. B.W.B. has received honoraria for speaking engagements and has given paid expert testimony in service to courts for his expertise in viral immunology. The other authors declare no conflicts of interest. The funder had no role in the design of the study; in the collection, analyses, or interpretation of data; in the writing of the manuscript; or in the decision to publish the results.

## Supporting information


Data S1.

## Data Availability

The data that support the findings of this study are available from the corresponding author upon reasonable request.
