# Supplementary material for: Characterisation and Sensitivity of a Canine Mast Cell Tumour Line to Oncolytic Viruses
Source: Vet Comp Oncol. 2024 Nov 11;23(1):42–51. doi: 10.1111/vco.13024 (PMC11830461; doi:10.1111/vco.13024)
Supplement: Supplementary file 1 — Data S1. [file VCO-23-42-s001.docx]

Article

**Supplementary Information**

**Material and methods**

**MCT-1** **Cell Seeding Assay**

A cell seeding assay was completed to determine optimal seeding density for the MCT-1 cell line in 96-well plates. To accomplish this, multiple cell densities were seeded into the wells of a 96-well plate. The densities began at 100,000 cells/well and decreased by a factor of two to generate a curve from which the optimal density for the cell line could be determined. Following a 48-hour incubation at 37˚C, resazurin sodium salt (Sigma Aldrich) was added. Fluorescence was quantified with a plate reader (excitation wavelength: 535/25 nm, emission wavelength: 590/35 nm) 3, 4, 5, and 6 hours after the addition of the resazurin (Supplementary Figure 1). The density corresponding to 90% of the maximum fluorescence was chosen for the resazurin assay to ensure maximum sensitivity of the assay while avoiding overgrowth of cells. This was determined to be 8,458 cells/well.

**Results**

**Optimization of MCT-1 Cell Seeding Density for Resazurin Assays**

**Supplementary Figure S1. The optimal cell seeding density calculation for MCT-1 cells.** The cells were seeded into wells of a 96-well plate at nine different seeding densities: 100000, 50000 , 25000, 12500, 6250, 3125, 1562.5, 781.25, 390.625. The plate was assessed at 3, 4, 5, and 6 hours after the addition of resazurin on a microplate reader. The density that corresponded to 90% of the maximum fluorescence was chosen for the resazurin assay. This was 8,458 cells.

**Supplementary Table 1. The optimal cell seeding density calculation for MCT-1 cells.**

A two-way analysis of variance with Dunnett's post-hoc multiple comparisons test was utilized for statistical analysis of the oncolytic effects of the viruses (rVSV-hDCT, ORFV, mesogenic AOaV-1, lentogentic AOaV-1, or rVSVΔm51). Shown are the p-values for the comparison of each oncolytic virus at nine different MOIs to untreated MCT-1 cells.

| **Table showing the results of a two-way analysis of variance** | | |
| --- | --- | --- |
| **Dunnett's multiple comparisons test** | **Summary** | **Adjusted *P* Value** |
| **rVSVΔm51** |  |  |
| Control vs. MOI 10 | **** | <0.0001 |
| Control vs. MOI 4 | **** | <0.0001 |
| Control vs. MOI 1.6 | **** | <0.0001 |
| Control vs. MOI 0.64 | **** | <0.0001 |
| Control vs. MOI 0.25 | **** | <0.0001 |
| Control vs. MOI 0.1 | **** | <0.0001 |
| Control vs. MOI 0.04 | **** | <0.0001 |
| Control vs. MOI 0.01 | **** | <0.0001 |
| Control vs. MOI 0.006 | **** | <0.0001 |
| **ORFV** |  |  |
| Control vs. MOI 10 | **** | <0.0001 |
| Control vs. MOI 4 | **** | <0.0001 |
| Control vs. MOI 1.6 | *** | 0.0002 |
| Control vs. MOI 0.64 | ** | 0.0016 |
| Control vs. MOI 0.25 | * | 0.037 |
| Control vs. MOI 0.1 | ns | 0.6035 |
| Control vs. MOI 0.04 | ns | 0.3709 |
| Control vs. MOI 0.01 | ns | 0.8224 |
| Control vs. MOI 0.006 | ns | 0.9919 |
| **AOaV-1-Mesogenic** |  |  |
| Control vs. MOI 10 | ** | 0.0072 |
| Control vs. MOI 4 | *** | 0.0001 |
| Control vs. MOI 1.6 | *** | 0.0007 |
| Control vs. MOI 0.64 | * | 0.0249 |
| Control vs. MOI 0.25 | ns | 0.0694 |
| Control vs. MOI 0.1 | ns | 0.1736 |
| Control vs. MOI 0.04 | ns | 0.8873 |
| Control vs. MOI 0.01 | ns | 0.8244 |
| Control vs. MOI 0.006 | ns | 0.9995 |
| **AOaV-1-Lentogenic** |  |  |
| Control vs. MOI 10 | **** | <0.0001 |
| Control vs. MOI 4 | **** | <0.0001 |
| Control vs. MOI 1.6 | **** | <0.0001 |
| Control vs. MOI 0.64 | **** | <0.0001 |
| Control vs. MOI 0.25 | ** | 0.0098 |
| Control vs. MOI 0.1 | ns | 0.4595 |
| Control vs. MOI 0.04 | ns | 0.6089 |
| Control vs. MOI 0.01 | ns | 0.9685 |
| Control vs. MOI 0.006 | ns | 0.9997 |

| **Table Showing Results of a Two-Way Analysis of Variance** | | |
| --- | --- | --- |
| **Tukey's multiple comparisons test** | **Summary** | **Adjusted *P*-Value** |
| **VSV vs. OrfV** | **** | <0.0001 |
| **VSV vs. AOaV-1-Mesogenic** | **** | <0.0001 |
| **VSV vs. AOaV-1-Lentogenic** | **** | <0.0001 |
| **OrfV vs. AOaV-1-Mesogenic** | ns | 0.2343 |
| **OrfV vs. AOaV-1-Lentogenic** | ns | 0.3976 |
| **AOaV-1-Mesogenic vs. AOaV-1-Lentogenic** | ** | 0.0044 |

**Supplementary Table 2. Differential Viral Oncolytic Efficacy in a Cell Line Derived from a MCT-1.**  Statistical analysis for figure 3 was performed using a two-way analysis of variance with Tukey’s multiple comparison test to compare the overall mean column effect among the treatment groups. *P*-values for the comparisons between oncolytic viruses are shown (n=3/treatment group).
